# Analogies of human speech and bird song: From vocal learning behavior to its neural basis

**DOI:** 10.3389/fpsyg.2023.1100969

**Published:** 2023-02-22

**Authors:** Yutao Zhang, Lifang Zhou, Jiachun Zuo, Songhua Wang, Wei Meng

**Affiliations:** Jiangxi Key Laboratory of Organic Chemistry, Jiangxi Science and Technology Normal University, Nanchang, China

**Keywords:** vocal learning, neural pathways, human language, bird song, analogy

## Abstract

Vocal learning is a complex acquired social behavior that has been found only in very few animals. The process of animal vocal learning requires the participation of sensorimotor function. By accepting external auditory input and cooperating with repeated vocal imitation practice, a stable pattern of vocal information output is eventually formed. In parallel evolutionary branches, humans and songbirds share striking similarities in vocal learning behavior. For example, their vocal learning processes involve auditory feedback, complex syntactic structures, and sensitive periods. At the same time, they have evolved the hierarchical structure of special forebrain regions related to vocal motor control and vocal learning, which are organized and closely associated to the auditory cortex. By comparing the location, function, genome, and transcriptome of vocal learning-related brain regions, it was confirmed that songbird singing and human language-related neural control pathways have certain analogy. These common characteristics make songbirds an ideal animal model for studying the neural mechanisms of vocal learning behavior. The neural process of human language learning may be explained through similar neural mechanisms, and it can provide important insights for the treatment of language disorders.

## 1. Introduction

Vocal learning is a rare animal behavior that learns to replicate conspecific or heterologous sounds or even artificial sounds through a process of repetitive neural activity from auditory to vocal motor (Janik and Slater, 2000; Jarvis, 2007). The role of vocal learning is to communicate sound information between individuals, including conspecific recognition, information transmission, deceptive hunting, etc. (Carouso-Peck et al., 2021). Human language was once thought to be the single most unique form of complex vocal learning behaviors among all animals. With the development of research, it has been proved that a few mammals and some birds (typically songbirds) have vocal learning behaviors similar to human speech (Le Boeuf and Peterson, 1969; Ralls et al., 1985; Poole et al., 2005; Jarvis, 2007; Janik, 2014).

Although mammals and birds evolved from different sources, there is growing evidence that the vocal learning processes of the two species are highly similar (Jarvis, 2004, 2019; Pfenning et al., 2014; Gedman et al., 2022). A comparison of neural control brain regions and pathways associated with vocal learning in mammals and birds (mainly songbirds), has led to the gradual emergence of evolutionary pathways of vocal learning behavior across species (Jarvis, 2004; Bolhuis et al., 2010; Lipkind et al., 2013). We define the similarity between songbird song and human speech from vocal learning behavior to its neural basis as analogies. By comparing the location, function and gene expression profiles of the brain regions, it is suggested that the songbird song control pathway and human language related pathway have certain analogy (Jarvis, 2007; Pfenning et al., 2014; Gedman et al., 2022). In addition, songbird song and human speech also show convergent evolution features, which makes the complexity of vocal learning in songbirds and humans comparable (Corballis, 2009; White, 2010). Therefore, songbirds become an ideal model for the study of vocal learning behavior, and can provide an important reference for studying the mechanisms of human language acquisition and the treatment of language disorders. In this article, we provide an overview of vocal learning behavior and neural control pathways in animals, especially the evolutionary analogy between human language and songbird song.

## 2. Vocal learning behavior in different animals

### 2.1. Types of animal vocalizations

Animal vocalizations can be divided into two types. One is the innate call, such as rooster crowing, human laughter and crying, and so on, which are controlled by species-specific vocal motor nuclei in the brainstem (including the midbrain and medulla oblongata) without the involvement of auditory feedback. All vocal vertebrates, from fish, amphibians, reptiles, birds to mammals, including humans, share similar brainstem vocal control pathways (Jarvis, 2007; Vergne et al., 2009; Feng and Bass, 2016; Kelley et al., 2020). The other type of vocalizations is produced through acquired vocal learning, which is produced by specific vocal control structures in the forebrain *via* neural projections to regulate brainstem vocal motor nuclei. Two parallel neural pathways are responsible for forebrain regulation of vocalization: the limbic vocal control pathway controls innate non-verbal and emotional vocalization; the laryngeal motor cortical pathway regulates fine motor control of voluntary vocalization, such as speaking and singing, as well as the spontaneous production of innate vocalization (Ludlow, 2005; Simonyan and Horwitz, 2011).

### 2.2. Vocal learning behavior in mammals, including humans

Species with different vocal learning behaviors differ greatly in their ability to imitate and modify sounds. For example, small mammal bats can use complex articulation including isolated calls, courtship calls, and territorial calls to promote echolocation and social behavior, and can adapt echolocation and social calls containing individual and gender information to their social environment (Vernes, 2017). Some large mammals also have the ability of vocal learning, for example, elephants, sea lions, and seals that had been kept in captivity for a long time can learn simple human language (Le Boeuf and Peterson, 1969; Ralls et al., 1985; Poole et al., 2005). In addition, among marine mammals, cetacean calls, and dolphin whistles have their own specific frequencies, and their acoustic signals are used to maintain contact between individuals when they are separated (Janik, 2014). Recent research on one particular species of rodents has shown that naked mole-rats (*Heterocephalus glaber*) from different regions can produce sounds with unique group information, similar to dialects (Barker et al., 2021). More evidence is needed, of course, to prove whether naked mole-rats are capable of vocal learning.

In primates, human vocal learning is undoubtedly the most complex and one of the most important behavioral bases of human language (Tyack, 2020). Through vocal learning, humans can imitate individual and continuous sounds and adjust the pronunciation by auditory feedback system (Tyack, 2020). Thus, human language is an auditory-directed vocal learning behavior, which is a hallmark function that distinguishes humans and other vocal learners from vocal non-learning animals, including primates (Hurford, 2003). However, the evolution of human language does not appear to have involved any single evolutionary mechanism unique to humans (Locke and Bogin, 2006). At the same time, human language, including spoken and signed language, can be regarded as a gesture system, i.e., a way of communicating specific information through visible body and hand movements (Liebal and Call, 2012). In fact, primate gesture systems are so well developed that they can generate and perceive hand movements. Therefore, human language is thought to have evolved from the gesture system, in which simple words were expressed by gestures at the beginning, but as communication became more frequent, complex spoken words replaced gestures as a new form of communication (Rizzolatti and Craighero, 2004). This hypothesis about the gesture system is consistent with Jarvis’s hypothesis that spoken language and sign language are equivalent to speech and signing respectively (Jarvis, 2019).

### 2.3. Similarities between bird song and human language

**FIGURE 1:**
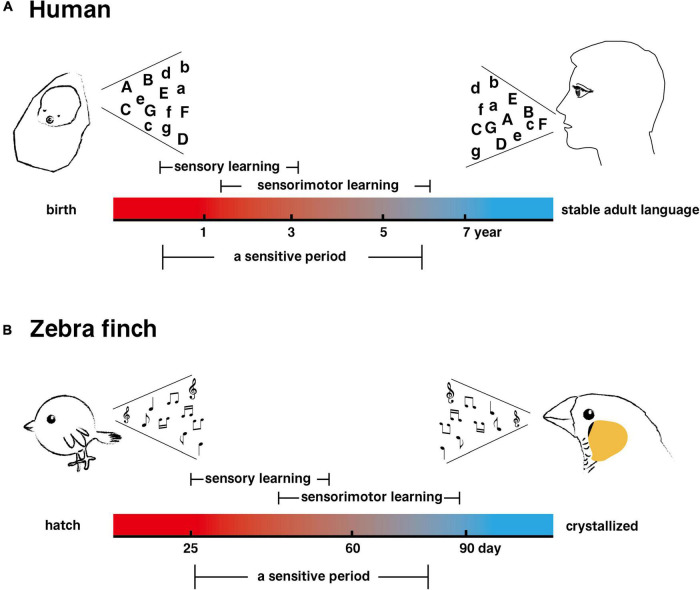
A comparison between the learning process of human speech and that of songbird singing. **(A)** The process of human speech learning. **(B)** The song learning process of a male zebra finch.

Research in the 1950s established that bird singing is a learned behavior (Nottebohm, 2014). Young birds need to imitate and practice the parent bird songs to form their own songs with complex acoustic characteristics. Birds with vocal learning behaviors, including parrots, songbirds, and hummingbirds, especially songbirds, whose songs are mainly for territorial defense and courtship behaviors (Thoms and Jürgens, 1987; Langmore, 1998; Rogers et al., 2006), have been widely used as model animals for studying the neural mechanisms of learned vocalization (Kao et al., 2005). A songbird’s song usually consists of several syllables, which form a fixed or variable pattern of syllable combinations (Mooney, 2022). The process of song learning in songbird juveniles is similar to that of human language learning in human infants (Figure 1), which also requires the participation of auditory feedback. It can be divided into sensory stage (storing the learned song or language in the brain through the interaction between innate factors and the environment) and sensorimotor stage (refining the template song or language for output) (Prather et al., 2017).

Moreover, the acquisition of the ability of auditory-vocal learning in both humans and songbirds occurs most rapidly during a critical early juvenile stage, the sensitive period (Doupe and Kuhl, 1999; Brainard and Doupe, 2002). The best time to learn their mother tongue is when human children are between 6 and 12 months old and begin to understand the external language and learn pronunciation (Hurford, 1991). In the case of songbirds, such as zebra finches (*Taeniopygia guttata*), juveniles learn the songs of their relatives during the sensitive period and gradually develop their own personalized and lifelong repertoire (Prather et al., 2017). Another important feature of human language is flexible control over complex syntactic structures, such as the repeated reordering of a set of words. Another songbird, Bengalese finches (*Lonchura striata domestica*), has the ability to control the ordering of syllables, which is similar to human control of the syntactic structure of language (Veit et al., 2021). According to these features, songbird song behavior has a high degree of similarity with human language function. Surprisingly, a recent study found that Australian musk ducks (*Biziura lobata*), a member of the Anseriformes family, also have vocal learning behavior, which provides more diversified information for deciphering the evolution of human language (Ten Cate and Fullagar, 2021).

## 3. Neural structures of controlling vocal learning behavior

The neural control of vocal learning behaviors does not rely on a single pathway or even a single brain region but is accomplished through the collaboration of related brain regions forming different pathways.

### 3.1. Neural control pathways of vocal learning behavior in mammals, including humans

#### 3.1.1. The limbic vocal control pathway

**FIGURE 2:**
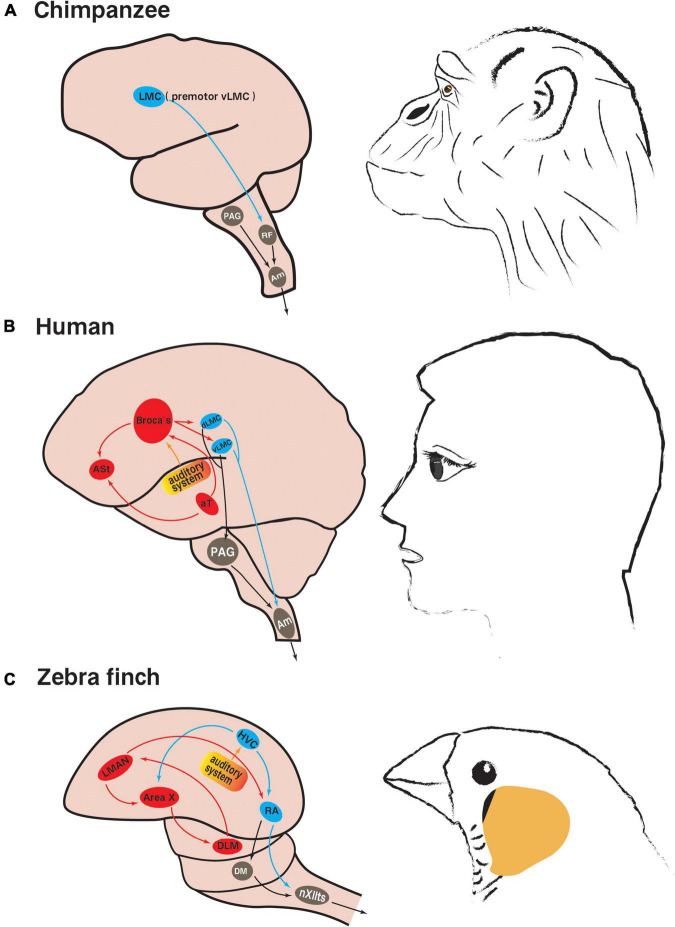
Neural pathways involved in innate vocalization and vocal learning. **(A)** Vocalization-related pathways in non-human primate chimpanzees (based on Kaas, 2012; Simonyan, 2014): the limbic vocal control pathway in gray; the laryngeal motor cortex pathway in blue. **(B)** Vocalization-related pathways in humans (based on Jarvis, 2007; Simonyan, 2014; Neef et al., 2021): the limbic vocal control pathway in gray; the laryngeal motor cortical pathway in blue; the language learning pathway in red. **(C)** Vocalization-related pathways in songbird zebra finches (based on Nottebohm, 1991; Jarvis, 2004): the innate brainstem vocal pathway in gray; the vocal motor pathway in blue; the anterior forebrain pathway related to song learning in red. The brain regions of the same color in songbirds and humans are analogous. The yellow part is the auditory system. LMC, laryngeal motor cortex; dLMC, dorsal LMC; vLMC, ventral LMC; RF, reticular formation; PAG, periaqueductal gray; Am, nucleus ambiguus; ASt, anterior striatum; aT, anterior thalamus speech area; HVC, used as a proper name; RA, robust nucleus of the arcopallium; LMAN, lateral part of the magnocellular nucleus of the anterior neostriatum; DLM, medial portion of the dorsolateral nucleus of the anterior thalamus; DM, dorsal medial midbrain nucleus; nXIIts, tracheosyringeal part of hypoglossal nucleus.

The limbic vocal control pathway in mammals, including primates, mainly controls innate vocalizations such as calls, crying and laughter. The periaqueductal gray (PAG) plays a central role in this pathway, as evidenced by the fact that damage to PAG results in complete vocal inability in cats, monkeys and humans (Adametz and O’Leary, 1959; Jürgens and Pratt, 1979; Esposito et al., 1999). PAG receives strong projections from the limbic system, anterior cingulate, insula, and orbitofrontal cortex. At the same time, PAG has strong projections dominating the nucleus ambiguus (Am) (Figures 2A, B). Am is the only motor neuron group that is directly involved in vocalization and can innervate the soft palate, pharynx, larynx, and diaphragm, intercostal muscles and abdominal muscles, which determine the intra-abdominal, intrathoracic, and subglottic pressures, and the control of these pressures is necessary to vocalization (Holstege and Subramanian, 2016).

#### 3.1.2. The laryngeal motor cortical pathway

In order to combine individual articulation into sentences, human speech requires the involvement of the laryngeal motor cortical pathway. The human laryngeal motor cortex (LMC), located ventral to the primary motor cortex, is responsible for fine motor control of voluntary vocalization such as speech and singing, as well as regulating the spontaneous production of innate vocalization (Simonyan and Horwitz, 2011). Natural and fluent speech requires flexible control of pitch and pronunciation. In humans, this voice control function is distributed in two LMC subregions of each hemisphere, the dorsal LMC (dLMC, located between the cortical representations of the lips and the hands) and the ventral LMC (vLMC, occupying parts of the subcentral gyrus and the rolandic operculum) (Bouchard et al., 2013; Pfenning et al., 2014; Neef et al., 2021). In particular, the tone modulation of speaking and singing is thought to be mainly controlled by dLMC (Dichter et al., 2018). Studies of persistent stuttering symptoms have shown that the cause of speech fluency disorder is the loss of white matter in the left vLMC, resulting in the separation of vLMC and the left lateral language area (Sommer et al., 2002), which indicates that vLMC mainly controls the fluency of speech, and further supports the conclusion that vLMC is functionally separate from dLMC (Neef et al., 2021).

The laryngeal motor cortical pathway is directed from dLMC/vLMC to Am, which coordinates laryngeal muscle movements and respiratory rhythm to precisely control vocalization (Iwatsubo et al., 1990). dLMC/vLMC can also project to PAG and indirectly send commands to Am through the limbic vocal control pathway (Figure 2B; Simonyan and Horwitz, 2011). If bilateral LMC is damaged or diseased, it will make the patient unable to speak and sing, but does not affect non-verbal vocalization, such as crying and laughing (Jürgens, 2002), indicating that LMC is not essential for the production of innate vocalizations, but is critical to human spoken vocalization. Unlike human LMC, which is located in the primary motor cortex, non-human primate LMC is located in the area 6 of premotor cortex (it is proposed to be premotor vLMC, similar to human vLMC) (Simonyan, 2014). This difference deserves special attention and may represent the evolutionary direction toward voluntary vocalization in humans (Simonyan and Horwitz, 2011).

In primates, another connection between LMC and the limbic vocal control pathway exists at the brainstem reticular formation (RF) (Figure 2A), particularly in the dorsal and parvocellular reticular nuclei of RF, which further forms direct connections with laryngeal motor neurons in Am, joint motor neurons in the trigeminal motor nucleus, the facial nucleus, the hypoglossal nucleus, and expiratory motor neurons in the thoracic and upper lumbar spinal cord (Thoms and Jürgens, 1987). Because the lack of direct projections from LMC to Am in non-human primates reduces the ability to directly modulate the activity of brainstem laryngeal motor neurons, the functional properties of RF are more important to vocal motor control in non-human primates than in humans (Iwatsubo et al., 1990).

#### 3.1.3. Human language learning pathway

In addition to the vocal motor pathway (VMP), human language learning, including the memory of vocabulary and grammar, relies on an additional forebrain pathway, the cortex-striatum-thalamus loop, consisting mainly of motor language center Broca’s area (in the posterior half of the left inferior frontal gyrus), the anterior striatum (ASt) and the anterior thalamus speech area (aT) (Figure 2B), and this language learning pathway is considered to be unavailable to non-human primates (Buckner et al., 1999; Jarvis, 2004; Gajardo-Vidal et al., 2021).

Voluntary production of words and sentences through the motor cortex requires a large amount of memory and involves the activity of a large number of neurons, many of which are located in the Broca’s area (Holstege and Subramanian, 2016). Broca’s area is responsible for language acquisition and high-level spoken language function, plays an important role in understanding and producing complex grammar and other language functions, and is a key node for manipulating and transmitting neural information in the large cortical network responsible for key components of language generation (Davis et al., 2008). Broca’s area is associated with several linguistic processes, including syntactic processing and unification, which involve the segmentation and concatenation of different types of linguistic information (Burton et al., 2000; Friederici, 2002). Although reading and repeating individual words does not involve semantic and syntactic processing, it does require the association of syllable sequences and motion gestures. Studies have shown that this association is coordinated by the interactions between Broca’s area and the temporal cortex, which processes auditory information, and the frontal cortex, which is responsible for motor function (Flinker et al., 2015). And Broca’s area is interconnected with LMC, so LMC receives instructions from Broca’s area (Flinker et al., 2015). However, it was recently reported that damage to Broca’s area alone does not affect long-term speech production after left frontal stroke, whereas that persistent speech production impairments can result from co-damage to Broca’s area and its adjacent white matter (Gajardo-Vidal et al., 2021).

Meanwhile, as a core component of human motor skill learning, ASt receives signal input from Broca’s area and remains activated for learning new words during the process of learning mother tongue in early childhood and second language in adulthood, indicating that ASt plays a key role in the process of language learning and memory (Simmonds et al., 2014). And the role of aT in speech, in addition to affecting the clarity of expression, may involve the mutual coordination of respiration and speech production (Bhatnager et al., 1989). Interestingly, the thalamus showed increased activity of predominantly left-sided neurons in response to language (Gogolitsin and Nechaev, 1990), consistent with the left-sided brain characteristic of human language.

It is traditionally believed that the superior temporal regional cortex (the sensory language center Wernicke’s area and its surrounding areas) is involved in the perception and memory of speech (Viceic et al., 2006). Although non-human primates also share homologous Broca-like and Wernicke-like areas with humans, damaging Broca-like area of monkeys and chimpanzees does not affect vocalization. The main reason is that their calls rely on the limbic vocal control pathway, rather than Broca-like area, which serves to understand gestures and facial emotions (Graïc et al., 2020). Meanwhile, Wernicke’s area supports the brain to understand articulatory phonemes and sequences, a process necessary for language production, including repetition of pronunciation, word extraction and reading aloud (Binder, 2015). Moreover, understanding the grammatical relations of words in sentences is fundamental to human language and unique to humans (Hauser et al., 2002; Marslen-Wilson and Tyler, 2007).

The differences between human and other primates in the language system are not only in the function of brain regions, but also in the location of the vocal organ, i.e., the larynx. In early childhood, the position of the larynx in humans is not much different from that of chimpanzees, but the human larynx rapidly descends to neck in early juvenile life. The descending position of the larynx contributes to the development of the human respiratory and digestive tracts and the formation of language function (Nishimura, 2005; Nishimura et al., 2008). However, this may not be the main reason why chimpanzees and other primates do not have human vocal learning behavior, as the decline of the larynx is not unique to humans, and the main reason may still be differences in brain structure, rather than differences in the anatomy of vocal organs (Fitch et al., 2016; Boë et al., 2017; Fitch, 2018; Jarvis, 2019).

### 3.2. Neural control pathways of avian vocal learning and analogy with human

#### 3.2.1. Neural control of instinctive vocalization in birds

The instinctive vocalization of all birds relies on a mammalian-like brainstem vocal control pathway, from the dorsomedial nucleus of the intercollicular complex (DM) in the midbrain to the tracheosyringeal part of hypoglossal nucleus (nXIIts) in the brainstem (Figure 2C), similar to the primate midbrain PAG and brainstem Am, respectively (Wild et al., 1997; Jarvis, 2004). The downstream projection pattern of DM in non-vocal learning birds and vocal learning birds is consistent with its role in respiratory-vocal regulation, and its neurons may project to both nXIIts vocal motor neurons and respiratory premotor neurons to realize the coordination between vocalization and respiration (Wild et al., 1997).

#### 3.2.2. Vocal learning pathways in songbirds similar to humans

Comparative studies of gene expression pattern of adult animals and molecular embryology indicate that avian and mammalian brains share an analogous cortex-basal ganglia-thalamus-cortex circuit associated with vocal learning behavior (Reiner et al., 2004; Jarvis et al., 2005). It is suggested that the avian and mammalian vocal behaviors have similar neural structural basis. Hummingbirds show complex vocal abilities in social activities, but so far there is no in-depth research on their vocal learning (Ferreira et al., 2006; Duque and Carruth, 2022). Parrots also have the ability to learn vocalizations, but knowledge of their learning process is still limited (Ten Cate, 2021). However, the richness of songbird species (passerine birds, about 4,000 species), the diversity of their vocal learning characteristics and the convenience of breeding and captivity make songbirds the most well-studied branch of birds for vocal learning behavior (Ten Cate, 2021). The distinctive birdsong of white-crowned sparrows (*Zonotrichia leucophrys*) was first described by Marler and Tamura (1964). It was not until the 1970s that Nottebohm et al. (1976) from Rockefeller University discovered the neural pathways related to song vocalization and learning in the brain of canaries (*Serinus canari*a).

Pfenning et al. (2014) compared the gene expression profiles of zebra finches and humans, showing that the telencephalon of songbirds is similar to the telencephalon of humans, and avian telencephalic subdivisions are similar to different subdivisions in mammals, and the brainstem nuclei of songbirds also correspond to the brainstem nuclei of humans. In the telencephalon, the cortex of songbirds closely resembles the human cerebral cortex, and the striatum of songbirds corresponds to the human striatum. Surprisingly, much of the neurobiological knowledge of human vocal learning has been inferred from the studies of songbirds (Saito and Maekawa, 1993; Doupe and Kuhl, 1999; Jarvis, 2004; Simmonds, 2015). Most of the vocal control nuclei in songbirds are located in the cortex, and two nuclei are located in the striatum and thalamus respectively, forming two interrelated song control pathways. One is the VMP, the other is the anterior forebrain pathway (AFP), collectively known as the song control system (Figure 2C).

#### 3.2.3. Analogy of vocal motor pathways in songbirds and humans

The accurate song of songbirds depends on the regulation of VMP, which consists of the song premotor nucleus HVC (used as a proper name) and the robust nucleus of the arcopallium (RA) in the telencephalon and nXIIts in the brainstem (Figure 2C; Marler and Doupe, 2000). HVC is not only the initiating brain region of VMP, but also the main input source of AFP, which is responsible for encoding the motif song, concurrently receives input from the auditory system and respectively transmits the integrated auditory information to RA and the striatal song control nuclei of AFP (Yu and Margoliash, 1996). These functions are performed by two groups of neurons within HVC that project to RA and striatum respectively. They play different roles in encoding song or regulating vocal plasticity, and their corresponding neural activity characteristics during singing are also different (Hessler and Okanoya, 2018). The observation of local field potential (LFP) signals in male zebra finches during singing indicates that the characteristic changes of time frequency structure of HVC LFP may correspond to specific syllables in the motif song. In addition, the HVC LFP signal features are similar to those LFP signals associated with motor control in mammals, including humans and non-human primates (Brown et al., 2021). Language-related premotor neural activity was found early in the human Broca’s area by electrophysiological recordings (Fried et al., 1981; Jarvis, 2004), while this area also receives signal input from the temporal auditory cortex and transmits the integrated auditory information to LMC and ASt, respectively (Doupe and Kuhl, 1999; Bolhuis and Gahr, 2006; Bolhuis et al., 2010). The idea that songbird HVC shares some similarities with human Broca’s area has thus been partially accepted. However, the comparison of lesion experiments suggests that the cortical nucleus in the songbird AFP is more analogous to human Broca’s area (Jarvis, 2004). Recent results of cellular transcriptomics further revealed the evolutionary features of songbird VMP. Although HVC and RA are not homologous with the mammalian neocortex, their similarity in cell types and connection mode suggests that VMP may have evolved to functionally resemble the mammalian neocortex (Colquitt et al., 2021). With the further study, the results of gene expression lineage analysis showed that the types of songbird HVC neurons are similar to those of human LMC layers 2–3, and human LMC layers 2–3 neurons project to LMC layer 5, just like songbird HVC neurons project to RA (Pfenning et al., 2014; Jarvis, 2019; Gedman et al., 2022).

RA is another major song premotor nucleus in the songbird forebrain and encodes important acoustic features of birdsongs (Sizemore and Perkel, 2008). RA is also the intersection nucleus of VMP and AFP, which integrates and encodes the input information from the superior nucleus HVC and AFP into the downstream nucleus nXIIts, and regulates the syringeal muscles and respiratory muscles to produce song behavior (Simonyan and Horwitz, 2011). RA dorsal neurons project to DM and modulate respiration and vocalization (Wild et al., 1997); RA ventral neurons project to nXIIts, which modulate syringeal muscle movements and ultimately control singing (Vicario, 1994). Functionally, both songbird RA and human LMC are vocal motor control brain regions, and damage to RA and LMC would cause both songbirds and humans to be unable to vocalize properly (Simonyan and Horwitz, 2011). Transcriptomic studies confirmed that songbird RA shares part of gene transcriptional profile with human LMC (Pfenning et al., 2014; Gedman et al., 2022). Further gene expression lineage alignment showed that the types of RA neurons are similar to those of human LMC layer 5 (Pfenning et al., 2014; Jarvis, 2019; Gedman et al., 2022). Recently, it has been reported that RA projection neurons exhibit electrophysiological features similar to those of specialized large pyramidal neurons in mammalian primary motor cortex, such as robust high-frequency firing, ultra-narrow spike waveforms, superfast Na^+^ current inactivation kinetics, and large resurgent Na^+^ currents (Zemel et al., 2021). In addition, it has been shown that the acoustic characteristics of learned song can be significantly affected by pharmacologically weakening or enhancing the activity of inhibitory interneurons in RA (Miller et al., 2017). This is similar to the extensive involvement of inhibitory interneurons in the regulation of motor planning and execution in the mammalian motor cortex (Merchant et al., 2012).

#### 3.2.4. Analogy of songbird song learning pathway and human language learning pathway

Songbirds also have a song learning pathway, AFP, which is similar to human language learning pathway and consists of the lateral part of the magnocellular nucleus of the anterior neostriatum (LMAN), the avian basal ganglia area X and the medial portion of the dorsolateral nucleus of the anterior thalamus (DLM) to form the cortex-basal ganglia-thalamus circuit (Figure 2C; Sizemore and Perkel, 2008). AFP is critical to birdsong plasticity, which modulates the effects of social signals on song behavior (Kao et al., 2008), and provides an ideal system for studying the role of cortex-basal ganglia circuit on experience-dependent skill learning (for example, mother tongue learning of infants) (Achiro et al., 2017).

Area X is a unique region in the basal ganglia of songbirds that is critical to song learning, which receives afferents from both LMAN of AFP and HVC of VMP, and is analogous to the mammalian striatum (Sasaki et al., 2006). All major physiological cell types found in the mammalian striatum exist in the avian area X, and both have nearly identical histochemical properties (Farries and Perkel, 2002). However, studies have shown that area X also contains neurons with the characteristics of the pallidum (Carrillo and Doupe, 2004). Two pallidal cell types in area X can be distinguished on the basis of singing-related neural activity, one of which is similar to thalamus-projecting neurons in the primate internal pallidal segment and the other is similar to non-thalamus-projecting neurons in the primate external pallidal segment (Goldberg et al., 2010). It has also been reported that the electrophysiological activities of two interneuron populations in area X, fast-spiking interneurons and external pallidal neurons, are different in response to the three behavioral states of non-singing, undirected singing and female-directed singing in male zebra finches, suggesting that social context may differentially modulate activity of multiple neuron types in area X (Woolley, 2016). The results of lesion experiments support the idea that songbird area X is functionally more similar to human ASt (Jarvis, 2007). Lately, it has been reported that damage to area X can cause lasting changes in cells and gene expression in its upstream and downstream nuclei, and may trigger neuroprotective mechanisms in the brain regions connected with it (Lukacova et al., 2022). Both songbird area X and human ASt are activated in response to the task demand of attempting completely novel articulatory motor sequences, and decline rapidly during the subsequent “habituation” process (Simmonds et al., 2014). However, songbird area X remains active after the adult birdsongs have stereotyped, which may be a difference from humans (Jarvis and Nottebohm, 1997; Hessler and Doupe, 1999; Simmonds et al., 2014). Furthermore, a recent analysis of genome-wide data of human rhythm and songbird vocal learning showed that several sets of genes associated with song behavior expressed in area X of zebra finches were significantly enriched in the gene structure of human beat synchronization, which supports the genetic and evolutionary correlation between the two rhythm-related behaviors, human beat synchronization and songbird singing (Gordon et al., 2021).

Cortical nucleus LMAN receives afferents from DLM and is the output nucleus of AFP that projects to RA of VMP (Luo et al., 2001), which is also a nucleus necessary for the song acquisition process of juvenile songbirds and plays a key role in adult songbirds producing different types of songs in different environments (Bottjer and Altenau, 2010; Achiro et al., 2017). It was found that when juvenile zebra finches are learning to sing, the firing patterns of individual neurons in core and shell subregions of LMAN were related to the acoustic similarity of learned tutor syllables, and the response variability of shell but not core subregion neurons decreased with the development and song learning process (Achiro et al., 2017). Moreover, damage to LMAN will result in a gradual decrease in the variability of songs, eventually becoming a single rigid song (Woolley et al., 2014). Jarvis (2004) suggested that in humans, not only Broca’s area but also the premotor LMC (preLMC) is involved in speech acquisition and advanced speech functions. Although the functional deficits caused by human preLMC damage are more complex, both the functional deficits caused by LMAN damage in songbirds and preLMC damage in humans result in reduced or even absent language imitation learning ability. In contrast, a recent study showed that enhancing the activity of LMAN can induce plastic changes in the acoustic structure of birdsongs, and cause singing repetitions and pauses similar to human stuttering symptoms (Chakraborty et al., 2017; Moorman et al., 2021).

DLM receives afferents from area X, and is analogous to the intralaminar nuclei of the mammalian thalamus (Nicholson et al., 2018), which is thought to be functionally similar to human aT, and is involved in the regulation of songbird song behavior (Jarvis, 2004). The electrophysiological properties of most DLM neurons are very similar to those of mammalian thalamocortical neurons, thus suggesting the conservation of thalamic neuron function in vertebrates (Luo and Perkel, 1999). Surprisingly, it was found that Bengalese finch DLM can also project to area X, suggesting that songbird area X receives feedback from thalamic regions while projecting to these regions, and further demonstrating the functional similarity between songbirds’ basal ganglia and mammalian basal ganglia (Nicholson et al., 2018). Damage to both songbird DLM and human anterior thalamus can lead to vocal behavior disorders, and in humans there is temporary silence followed by aphasia, sometimes more severe than damage to ASt or premotor cortex, probably due to further convergence of striatal inputs to the thalamus (Graff-Radford et al., 1985; Halsema and Bottjer, 1991).

## 4. Conclusion and prospect

In summary, the brainstem vocal control pathways that control innate vocalizations exist in almost every species of mammals and birds, but only a few species, including humans and some birds, possess vocal learning abilities and related neural pathways. Accidental discoveries of individual cases of animals imitating human speech, such as elephants, seals, and parrots, suggest that there may be other species’ vocal learning behaviors that have not yet been deciphered. Meanwhile, the differences of complex vocal behaviors and their neural control between mammals and birds predict that the formation of vocal learning behaviors in different species may have multiple independent origins (Tyack, 2020). In recent decades, it has been clearly understood that human beings and songbirds at a completely different evolutionary level have similar evolutionary paths of vocal behaviors. The results of studies at the level of genomics and transcriptomics suggest the potential analogy of neural pathways related to vocal learning between the two species.

The ethology of songbird song learning has been studied for decades from its inception to its interdisciplinary study with neurobiology. The anatomical structure of songbird song control system and its role in regulating song behavior have been comprehensively understood (Nottebohm, 2005; Jarvis, 2019). The effects of neurotransmitters, hormones, neurotrophins, and other bioactive substances on the song behavior of songbirds remain to be further studied (Meng et al., 2016, 2017; Tanaka et al., 2018; Wang et al., 2019, 2020; Jaffe and Brainard, 2020; Macedo-Lima and Remage-Healey, 2020; Miller et al., 2020; Zhang et al., 2022). Some cutting-edge technologies have pushed the field to a deeper level. Many speech disorders may be related to neurotransmitter signaling (Anderson et al., 1999; Craig-McQuaide et al., 2014). A study using a combination of optogenetics and gene manipulation techniques has shown that singing disorders in songbirds may be related to dopaminergic signaling in area X, which may be similar to the occurrence of language disorders (Xiao et al., 2021). However, it is still unclear how various neurotransmitters, hormones and neurotrophins regulate songbird singing behavior through related neural pathways. Optogenetics, chemogenetics and other targeted neural pathway manipulation techniques can be a key link between behavior and neural activity (Singh Alvarado et al., 2021). In the meantime, the related cell types and gene expression patterns of birds and mammals were compared by single-cell sequencing technology to reveal their evolutionary analogy (Colquitt et al., 2021). Commonly used songbird models were gene-edited using CRISPR/Cas9 technology to make them more widely applicable for multi-purpose studies (Biegler et al., 2022). The effects of experience and internal and external environment on the neurogenome or transcriptome of songbirds and their correlation with singing behavior were revealed by epigenomics studies (Kelly et al., 2018). These studies may be the focus of birdsong neurobiology.

More challengingly, how vocal learning changes with time and experience in different ages, the patterns of activity and association of relevant brain regions, and how auditory feedback plays a role are key issues that both human language and bird song research fields share and need to address (Doupe and Kuhl, 1999). However, many of the invasive experiments exploring the physiological mechanisms of vocalization, including language learning, cannot be performed in healthy humans. Given that avian song learning may share the underlying cellular and molecular regulatory mechanisms with human language learning, drawing on the research model of avian song behavior studies could shed light on the neural mechanisms of human language learning and the treatment of language disorders (Medina et al., 2022).

## Author contributions

YZ: investigation and writing—original draft. LZ: investigation. JZ: visualization. SW: funding acquisition. WM: conceptualization, validation, supervision, funding acquisition, and writing—review and editing. All authors contributed to the article and approved the submitted version.

## References

[B1] Achiro J. M. Shen J. Bottjer S. W. (2017). Neural activity in cortico-basal ganglia circuits of juvenile songbirds encodes performance during goal-directed learning. *Elife* 6:e26973. 10.7554/eLife.26973 29256393 PMC5762157

[B2] Adametz J. O’Leary J. L. (1959). Experimental mutism resulting from periaqueductal lesions in cats. *Neurology* 9 636–642. 10.1212/wnl.9.10.636 13791737

[B3] Anderson J. M. Hughes J. D. Rothi L. J. Crucian G. P. Heilman K. M. (1999). Developmental stuttering and Parkinson’s disease: the effects of levodopa treatment. *J. Neurol. Neurosurg. Psychiatry* 66 776–778. 10.1136/jnnp.66.6.776 10329754 PMC1736378

[B4] Barker A. J. Veviurko G. Bennett N. C. Hart D. W. Mograby L. Lewin G. R. (2021). Cultural transmission of vocal dialect in the naked mole-rat. *Science* 371 503–507. 10.1126/science.abc6588 33510025

[B5] Bhatnager S. C. Andy O. J. Korabic E. W. Tikofsky R. S. Saxena V. K. Hellman R. S. (1989). The effect of thalamic stimulation in processing of verbal stimuli in dichotic listening tasks: a case study. *Brain Lang.* 36 236–251. 10.1016/0093-934x(89)90063-1 2784070

[B6] Biegler M. T. Fedrigo O. Collier P. Mountcastle J. Haase B. Tilgner H. U. (2022). Induction of an immortalized songbird cell line allows for gene characterization and knockout by CRISPR-Cas9. *Sci. Rep.* 12:4369. 10.1038/s41598-022-07434-7 35288582 PMC8921232

[B7] Binder J. R. (2015). The Wernicke area: modern evidence and a reinterpretation. *Neurology* 85 2170–2175. 10.1212/wnl.0000000000002219 26567270 PMC4691684

[B8] Boë L. J. Berthommier F. Legou T. Captier G. Kemp C. Sawallis T. R. (2017). Evidence of a vocalic proto-system in the baboon (Papio papio) suggests pre-hominin speech precursors. *PLoS One* 12:e0169321. 10.1371/journal.pone.0169321 28076426 PMC5226677

[B9] Bolhuis J. J. Gahr M. (2006). Neural mechanisms of birdsong memory. *Nat. Rev. Neurosci.* 7 347–357. 10.1038/nrn1904 16760915

[B10] Bolhuis J. J. Okanoya K. Scharff C. (2010). Twitter evolution: converging mechanisms in birdsong and human speech. *Nat. Rev. Neurosci.* 11 747–759. 10.1038/nrn2931 20959859

[B11] Bottjer S. W. Altenau B. (2010). Parallel pathways for vocal learning in basal ganglia of songbirds. *Nat. Neurosci.* 13 153–155. 10.1038/nn.2472 20023650 PMC2846604

[B12] Bouchard K. E. Mesgarani N. Johnson K. Chang E. F. (2013). Functional organization of human sensorimotor cortex for speech articulation. *Nature* 495 327–332. 10.1038/nature11911 23426266 PMC3606666

[B13] Brainard M. S. Doupe A. J. (2002). What songbirds teach us about learning. *Nature* 417 351–358. 10.1038/417351a 12015616

[B14] Brown D. E. II Chavez J. I. Nguyen D. H. Kadwory A. Voytek B. Arneodo E. M. (2021). Local field potentials in a pre-motor region predict learned vocal sequences. *PLoS Comput. Biol.* 17:e1008100. 10.1371/journal.pcbi.1008100 34555020 PMC8460039

[B15] Buckner R. L. Kelley W. M. Petersen S. E. (1999). Frontal cortex contributes to human memory formation. *Nat. Neurosci.* 2 311–314. 10.1038/7221 10204536

[B16] Burton M. W. Small S. L. Blumstein S. E. (2000). The role of segmentation in phonological processing: an fMRI investigation. *J. Cogn. Neurosci.* 12 679–690. 10.1162/089892900562309 10936919

[B17] Carouso-Peck S. Goldstein M. H. Fitch W. T. (2021). The many functions of vocal learning. *Philos. Trans. R. Soc. Lond. B Biol. Sci.* 376:20200235. 10.1098/rstb.2020.0235 34482721 PMC8419581

[B18] Carrillo G. D. Doupe A. J. (2004). Is the songbird Area X striatal, pallidal, or both? An anatomical study. *J. Comp. Neurol.* 473 415–437. 10.1002/cne.20099 15116398

[B19] Chakraborty M. Chen L. F. Fridel E. E. Klein M. E. Senft R. A. Sarkar A. (2017). Overexpression of human NR2B receptor subunit in LMAN causes stuttering and song sequence changes in adult zebra finches. *Sci Rep* 7 942. 10.1038/s41598-017-00519-8 28432288 PMC5430713

[B20] Colquitt B. M. Merullo D. P. Konopka G. Roberts T. F. Brainard M. S. (2021). Cellular transcriptomics reveals evolutionary identities of songbird vocal circuits. *Science* 371:eabd9704. 10.1126/science.abd9704 33574185 PMC8136249

[B21] Corballis M. C. (2009). The evolution of language. *Ann. N.Y. Acad. Sci.* 1156 19–43. 10.1111/j.1749-6632.2009.04423.x 19338501

[B22] Craig-McQuaide A. Akram H. Zrinzo L. Tripoliti E. (2014). A review of brain circuitries involved in stuttering. *Front. Hum. Neurosci.* 8:884. 10.3389/fnhum.2014.00884 25452719 PMC4233907

[B23] Davis C. Kleinman J. T. Newhart M. Gingis L. Pawlak M. Hillis A. E. (2008). Speech and language functions that require a functioning Broca’s area. *Brain Lang.* 105 50–58. 10.1016/j.bandl.2008.01.012 18325581

[B24] Dichter B. K. Breshears J. D. Leonard M. K. Chang E. F. (2018). The control of vocal pitch in human laryngeal motor cortex. *Cell* 174 21–31.e9. 10.1016/j.cell.2018.05.016 29958109 PMC6084806

[B25] Doupe A. J. Kuhl P. K. (1999). Birdsong and human speech: common themes and mechanisms. *Annu. Rev. Neurosci.* 22 567–631. 10.1146/annurev.neuro.22.1.567 10202549

[B26] Duque F. G. Carruth L. L. (2022). Vocal communication in hummingbirds. *Brain Behav. Evol.* 97 241–252. 10.1159/000522148 35073546

[B27] Esposito A. Demeurisse G. Alberti B. Fabbro F. (1999). Complete mutism after midbrain periaqueductal gray lesion. *Neuroreport* 10 681–685. 10.1097/00001756-199903170-00004 10208530

[B28] Farries M. A. Perkel D. J. (2002). A telencephalic nucleus essential for song learning contains neurons with physiological characteristics of both striatum and globus pallidus. *J. Neurosci.* 22 3776–3787. 10.1523/jneurosci.22-09-03776.2002 11978853 PMC6758399

[B29] Feng N. Y. Bass A. H. (2016). “Singing” fish rely on circadian rhythm and melatonin for the timing of nocturnal courtship vocalization. *Curr. Biol.* 26 2681–2689. 10.1016/j.cub.2016.07.079 27666972

[B30] Ferreira A. R. Smulders T. V. Sameshima K. Mello C. V. Jarvis E. D. (2006). Vocalizations and associated behaviors of the sombre hummingbird (*Aphantochroa cirrhochloris*) and the rufous-breasted hermit (*Glaucis hirsutus*). *Auk* 123 1129–1148. 10.1642/0004-80382006123[1129:vaabot]2.0.co;2 18802498 PMC2542898

[B31] Fitch W. T. (2018). The biology and evolution of speech: a comparative analysis. *Annu. Rev. Linguist.* 4 255–279.

[B32] Fitch W. T. de Boer B. Mathur N. Ghazanfar A. A. (2016). Monkey vocal tracts are speech-ready. *Sci Adv* 2 e1600723. 10.1126/sciadv.1600723 27957536 PMC5148209

[B33] Flinker A. Korzeniewska A. Shestyuk A. Y. Franaszczuk P. J. Dronkers N. F. Knight R. T. (2015). Redefining the role of Broca’s area in speech. *Proc. Natl. Acad. Sci. U.S.A.* 112 2871–2875. 10.1073/pnas.1414491112 25730850 PMC4352780

[B34] Fried I. Ojemann G. A. Fetz E. E. (1981). Language-related potentials specific to human language cortex. *Science* 212 353–356. 10.1126/science.7209537 7209537

[B35] Friederici A. D. (2002). Towards a neural basis of auditory sentence processing. *Trends Cogn. Sci.* 6 78–84. 10.1016/s1364-6613(00)01839-8 15866191

[B36] Gajardo-Vidal A. Lorca-Puls D. L. Team P. Warner H. Pshdary B. Crinion J. T. (2021). Damage to Broca’s area does not contribute to long-term speech production outcome after stroke. *Brain* 144 817–832. 10.1093/brain/awaa460 33517378 PMC8041045

[B37] Gedman G. L. Biegler M. T. Haase B. Wirthlin M. E. Fedrigo O. Pfenning A. R. (2022). Convergent gene expression highlights shared vocal motor microcircuitry in songbirds and humans. *bioRxiv* [Preprint]. 10.1101/2022.07.01.498177

[B38] Gogolitsin Y. L. Nechaev V. B. (1990). Correlates of lexical processing in the activity of neuronal populations of the human brain. *Stereotact. Funct. Neurosurg.* 5 163–167. 10.1159/000100208 2080332

[B39] Goldberg J. H. Adler A. Bergman H. Fee M. S. (2010). Singing-related neural activity distinguishes two putative pallidal cell types in the songbird basal ganglia: comparison to the primate internal and external pallidal segments. *J. Neurosci.* 30 7088–7098. 10.1523/jneurosci.0168-10.2010 20484651 PMC2874984

[B40] Gordon R. L. Ravignani A. Hyland Bruno J. Robinson C. M. Scartozzi A. Embalabala R. (2021). Linking the genomic signatures of human beat synchronization and learned song in birds. *Philos Trans. R. Soc. Lond. B. Biol. Sci.* 376:20200329. 10.1098/rstb.2020.0329 34420388 PMC8380983

[B41] Graff-Radford N. R. Damasio H. Yamada T. Eslinger P. J. Damasio A. R. (1985). Nonhaemorrhagic thalamic infarction. Clinical, neuropsychological and electrophysiological findings in four anatomical groups defined by computerized tomography. *Brain* 108(Pt 2) 485–516. 10.1093/brain/108.2.485 4005533

[B42] Graïc J. M. Peruffo A. Corain L. Centelleghe C. Granato A. Zanellato E. (2020). Asymmetry in the cytoarchitecture of the area 44 homolog of the brain of the chimpanzee pan troglodytes. *Front. Neuroanat.* 14:55. 10.3389/fnana.2020.00055 32973465 PMC7471632

[B43] Halsema K. Bottjer S. (1991). Lesioning afferent input to a forebrain nucleus disrupts vocal learning in zebra finches. *Soc. Neurosci. Abstracts* 17:1052.

[B44] Hauser M. D. Chomsky N. Fitch W. T. (2002). The faculty of language: what is it, who has it, and how did it evolve? *Science* 298 1569–1579. 10.1126/science.298.5598.1569 12446899

[B45] Hessler N. A. Doupe A. J. (1999). Singing-related neural activity in a dorsal forebrain-basal ganglia circuit of adult zebra finches. *J. Neurosci.* 19 10461–10481. 10.1523/jneurosci.19-23-10461.1999 10575043 PMC6782438

[B46] Hessler N. A. Okanoya K. (2018). Physiological identification of cortico-striatal projection neurons for song control in Bengalese finches. *Behav. Brain Res.* 349 37–41. 10.1016/j.bbr.2018.04.044 29709609

[B47] Holstege G. Subramanian H. H. (2016). Two different motor systems are needed to generate human speech. *J. Comp. Neurol.* 524 1558–1577. 10.1002/cne.23898 26355872

[B48] Hurford J. R. (1991). The evolution of the critical period for language acquisition. *Cognition* 40 159–201. 10.1016/0010-0277(91)90024-x 1786674

[B49] Hurford J. R. (2003). The neural basis of predicate-argument structure. *Behav. Brain Sci.* 26 261–283; discussion 283–316. 10.1017/s0140525x03000074 14968690

[B50] Iwatsubo T. Kuzuhara S. Kanemitsu A. Shimada H. Toyokura Y. (1990). Corticofugal projections to the motor nuclei of the brainstem and spinal cord in humans. *Neurology* 40 309–312. 10.1212/wnl.40.2.309 2300253

[B51] Jaffe P. I. Brainard M. S. (2020). Acetylcholine acts on songbird premotor circuitry to invigorate vocal output. *Elife* 9 e53288. 10.7554/eLife.53288 32425158 PMC7237207

[B52] Janik V. M. (2014). Cetacean vocal learning and communication. *Curr. Opin. Neurobiol.* 28 60–65. 10.1016/j.conb.2014.06.010 25057816

[B53] Janik V. M. Slater P. J. (2000). The different roles of social learning in vocal communication. *Anim. Behav.* 60 1–11. 10.1006/anbe.2000.1410 10924198

[B54] Jarvis E. D. (2004). Learned birdsong and the neurobiology of human language. *Ann. N.Y. Acad. Sci.* 1016 749–777. 10.1196/annals.1298.038 15313804 PMC2485240

[B55] Jarvis E. D. (2007). Neural systems for vocal learning in birds and humans: a synopsis. *J Ornithol* 148 35–44. 10.1007/s10336-007-0243-0 19684872 PMC2726745

[B56] Jarvis E. D. (2019). Evolution of vocal learning and spoken language. *Science* 366 50–54. 10.1126/science.aax0287 31604300

[B57] Jarvis E. D. Nottebohm F. (1997). Motor-driven gene expression. *Proc. Natl. Acad. Sci. U.S.A.* 94 4097–4102. 10.1073/pnas.94.8.4097 9108111 PMC20574

[B58] Jarvis E. D. Güntürkün O. Bruce L. Csillag A. Karten H. Kuenzel W. (2005). Avian brains and a new understanding of vertebrate brain evolution. *Nat. Rev. Neurosci.* 6 151–159. 10.1038/nrn1606 15685220 PMC2507884

[B59] Jürgens U. (2002). Neural pathways underlying vocal control. *Neurosci. Biobehav. Rev.* 26 235–258. 10.1016/s0149-7634(01)00068-9 11856561

[B60] Jürgens U. Pratt R. (1979). Role of the periaqueductal grey in vocal expression of emotion. *Brain Res.* 167 367–378. 10.1016/0006-8993(79)90830-8 109167

[B61] Kaas J. H. (2012). The evolution of neocortex in primates. *Prog. Brain Res.* 195 91–102. 10.1016/b978-0-444-53860-4.00005-2 22230624 PMC3787901

[B62] Kao M. H. Doupe A. J. Brainard M. S. (2005). Contributions of an avian basal ganglia-forebrain circuit to real-time modulation of song. *Nature* 433 638–643. 10.1038/nature03127 15703748

[B63] Kao M. H. Wright B. D. Doupe A. J. (2008). Neurons in a forebrain nucleus required for vocal plasticity rapidly switch between precise firing and variable bursting depending on social context. *J. Neurosci.* 28 13232–13247. 10.1523/jneurosci.2250-08.2008 19052215 PMC3022006

[B64] Kelley D. B. Ballagh I. H. Barkan C. L. Bendesky A. Elliott T. M. Evans B. J. (2020). Generation, coordination, and evolution of neural circuits for vocal communication. *J. Neurosci.* 40 22–36. 10.1523/jneurosci.0736-19.2019 31896561 PMC6939475

[B65] Kelly T. K. Ahmadiantehrani S. Blattler A. London S. E. (2018). Epigenetic regulation of transcriptional plasticity associated with developmental song learning. *Proc. Biol. Sci.* 285:20180160. 10.1098/rspb.2018.0160 29720411 PMC5966592

[B66] Langmore N. E. (1998). Functions of duet and solo songs of female birds. *Trends Ecol. Evol.* 13 136–140. 10.1016/s0169-5347(97)01241-x 21238233

[B67] Le Boeuf B. J. Peterson R. S. (1969). Dialects in elephatn seals. *Science* 166 1654–1656. 10.1126/science.166.3913.1654 5360593

[B68] Liebal K. Call J. (2012). The origins of non-human primates’ manual gestures. *Philos. Trans. R. Soc. Lond. B. Biol. Sci.* 367 118–128. 10.1098/rstb.2011.0044 22106431 PMC3223783

[B69] Lipkind D. Marcus G. F. Bemis D. K. Sasahara K. Jacoby N. Takahasi M. (2013). Stepwise acquisition of vocal combinatorial capacity in songbirds and human infants. *Nature* 498 104–108. 10.1038/nature12173 23719373 PMC3676428

[B70] Locke J. L. Bogin B. (2006). Language and life history: a new perspective on the development and evolution of human language. *Behav. Brain Sci.* 29 259–280; discussion 280–325. 10.1017/s0140525x0600906x 17214017

[B71] Ludlow C. L. (2005). Central nervous system control of the laryngeal muscles in humans. *Respir. Physiol. Neurobiol.* 147 205–222. 10.1016/j.resp.2005.04.015 15927543 PMC1351146

[B72] Lukacova K. Hamaide J. Baciak L. Van der Linden A. Kubikova L. (2022). Striatal injury induces overall brain alteration at the pallial, thalamic, and cerebellar levels. *Biology* 11:425. 10.3390/biology11030425 35336799 PMC8945699

[B73] Luo M. Perkel D. J. (1999). A GABAergic, strongly inhibitory projection to a thalamic nucleus in the zebra finch song system. *J. Neurosci.* 19 6700–6711. 10.1523/jneurosci.19-15-06700.1999 10414999 PMC6782801

[B74] Luo M. Ding L. Perkel D. J. (2001). An avian basal ganglia pathway essential for vocal learning forms a closed topographic loop. *J. Neurosci.* 21 6836–6845. 10.1523/jneurosci.21-17-06836.2001 11517271 PMC6763103

[B75] Macedo-Lima M. Remage-Healey L. (2020). Auditory learning in an operant task with social reinforcement is dependent on neuroestrogen synthesis in the male songbird auditory cortex. *Horm. Behav.* 121:104713. 10.1016/j.yhbeh.2020.104713 32057821 PMC7198363

[B76] Marler P. Doupe A. J. (2000). Singing in the brain. *Proc. Natl. Acad. Sci. U.S.A.* 97 2965–2967. 10.1073/pnas.97.7.2965 10737777 PMC34308

[B77] Marler P. Tamura M. (1964). Culturally transmitted patterns of vocal behavior in sparrows. *Science* 146 1483–1486. 10.1126/science.146.3650.1483 14208581

[B78] Marslen-Wilson W. D. Tyler L. K. (2007). Morphology, language and the brain: the decompositional substrate for language comprehension. *Philos. Trans. R. Soc. Lond. B. Biol. Sci.* 362 823–836. 10.1098/rstb.2007.2091 17395577 PMC2430000

[B79] Medina C. A. Vargas E. Munger S. J. Miller J. E. (2022). Vocal changes in a zebra finch model of Parkinson’s disease characterized by alpha-synuclein overexpression in the song-dedicated anterior forebrain pathway. *PLoS One* 17:e0265604. 10.1371/journal.pone.0265604 35507553 PMC9067653

[B80] Meng W. Wang S. H. Li D. F. (2016). Carbachol-induced reduction in the activity of adult male zebra finch RA projection neurons. *Neural Plast.* 2016:7246827. 10.1155/2016/7246827 26904300 PMC4745321

[B81] Meng W. Wang S. Yao L. Zhang N. Li D. (2017). Muscarinic receptors are responsible for the cholinergic modulation of projection neurons in the song production brain nucleus RA of zebra finches. *Front. Cell Neurosci.* 11:51. 10.3389/fncel.2017.00051 28293176 PMC5329057

[B82] Merchant H. de Lafuente V. Peña-Ortega F. Larriva-Sahd J. (2012). Functional impact of interneuronal inhibition in the cerebral cortex of behaving animals. *Prog. Neurobiol.* 99 163–178. 10.1016/j.pneurobio.2012.08.005 22960789

[B83] Miller K. E. Wood W. E. Brenowitz E. A. Perkel D. J. (2020). Brain-derived neurotrophic factor has a transsynaptic trophic effect on neural activity in an adult forebrain circuit. *J. Neurosci.* 40 1226–1231. 10.1523/jneurosci.2375-19.2019 31857358 PMC7002143

[B84] Miller M. N. Cheung C. Y. J. Brainard M. S. (2017). Vocal learning promotes patterned inhibitory connectivity. *Nat. Commun.* 8:2105. 10.1038/s41467-017-01914-5 29235480 PMC5727387

[B85] Mooney R. (2022). Birdsong. *Curr. Biol.* 32 R1090–R1094. 10.1016/j.cub.2022.07.006 36283371 PMC11735021

[B86] Moorman S. Ahn J. R. Kao M. H. (2021). Plasticity of stereotyped birdsong driven by chronic manipulation of cortical-basal ganglia activity. *Curr. Biol.* 31 2619–2632.e4. 10.1016/j.cub.2021.04.030 33974850 PMC8222193

[B87] Neef N. E. Primaßin A. von Gudenberg A. W. Dechent P. Riedel H. C. Paulus W. (2021). Two cortical representations of voice control are differentially involved in speech fluency. *Brain Commun.* 3:fcaa232. 10.1093/braincomms/fcaa232 33959707 PMC8088816

[B88] Nicholson D. A. Roberts T. F. Sober S. J. (2018). Thalamostriatal and cerebellothalamic pathways in a songbird, the Bengalese finch. *J. Comp. Neurol.* 526 1550–1570. 10.1002/cne.24428 29520771 PMC5899675

[B89] Nishimura T. (2005). Developmental changes in the shape of the supralaryngeal vocal tract in chimpanzees. *Am. J. Phys. Anthropol.* 126 193–204. 10.1002/ajpa.20112 15386289

[B90] Nishimura T. Oishi T. Suzuki J. Matsuda K. Takahashi T. (2008). Development of the supralaryngeal vocal tract in Japanese macaques: implications for the evolution of the descent of the larynx. *Am. J. Phys. Anthropol.* 135 182–194. 10.1002/ajpa.20719 17960727

[B91] Nottebohm F. (1991). Reassessing the mechanisms and origins of vocal learning in birds. *Trends Neurosci.* 14 206–211. 10.1016/0166-2236(91)90107-6 1713723

[B92] Nottebohm F. (2005). The neural basis of birdsong. *PLoS Biol.* 3:e164. 10.1371/journal.pbio.0030164 15884976 PMC1110917

[B93] Nottebohm F. (2014). Peter marler (1928-2014). *Nature* 512:372. 10.1038/512372a 25164741

[B94] Nottebohm F. Stokes T. M. Leonard C. M. (1976). Central control of song in the canary, *Serinus canarius*. *J Comp Neurol* 165 457–486. 10.1002/cne.901650405 1262540

[B95] Pfenning A. R. Hara E. Whitney O. Rivas M. V. Wang R. Roulhac P. L. (2014). Convergent transcriptional specializations in the brains of humans and song-learning birds. *Science* 346:1256846. 10.1126/science.1256846 25504733 PMC4385736

[B96] Poole J. H. Tyack P. L. Stoeger-Horwath A. S. Watwood S. (2005). Animal behaviour: elephants are capable of vocal learning. *Nature* 434 455–456. 10.1038/434455a 15791244

[B97] Prather J. F. Okanoya K. Bolhuis J. J. (2017). Brains for birds and babies: neural parallels between birdsong and speech acquisition. *Neurosci. Biobehav. Rev.* 81(Pt. B) 225–237. 10.1016/j.neubiorev.2016.12.035 28087242

[B98] Ralls K. Fiorelli P. Gish S. (1985). Vocalizations and vocal mimicry in captive harbor seals, *Phoca vitulina*. *Can. J Zool.* 63 1050–1056.

[B99] Reiner A. Perkel D. J. Bruce L. L. Butler A. B. Csillag A. Kuenzel W. (2004). Revised nomenclature for avian telencephalon and some related brainstem nuclei. *J. Comp. Neurol.* 473 377–414. 10.1002/cne.20118 15116397 PMC2518311

[B100] Rizzolatti G. Craighero L. (2004). The mirror-neuron system. *Annu. Rev. Neurosci.* 27 169–192. 10.1146/annurev.neuro.27.070203.144230 15217330

[B101] Rogers A. C. Langmore N. E. Mulder R. A. (2006). Function of pair duets in the eastern whipbird: cooperative defense or sexual conflict? *Behav. Ecol.* 18 182–188. 10.1093/beheco/arl070

[B102] Saito N. Maekawa M. (1993). Birdsong: the interface with human language. *Brain Dev.* 15 31–39. 10.1016/0387-7604(93)90004-r 8338209

[B103] Sasaki A. Sotnikova T. D. Gainetdinov R. R. Jarvis E. D. (2006). Social context-dependent singing-regulated dopamine. *J. Neurosci.* 26 9010–9014. 10.1523/jneurosci.1335-06.2006 16943558 PMC2474783

[B104] Simmonds A. J. (2015). A hypothesis on improving foreign accents by optimizing variability in vocal learning brain circuits. *Front. Hum. Neurosci.* 9:606. 10.3389/fnhum.2015.00606 26582984 PMC4631821

[B105] Simmonds A. J. Leech R. Iverson P. Wise R. J. (2014). The response of the anterior striatum during adult human vocal learning. *J. Neurophysiol.* 112 792–801. 10.1152/jn.00901.2013 24805076 PMC4122740

[B106] Simonyan K. (2014). The laryngeal motor cortex: its organization and connectivity. *Curr. Opin. Neurobiol.* 28 15–21. 10.1016/j.conb.2014.05.006 24929930 PMC4177508

[B107] Simonyan K. Horwitz B. (2011). Laryngeal motor cortex and control of speech in humans. *Neuroscientist* 17 197–208. 10.1177/1073858410386727 21362688 PMC3077440

[B108] Singh Alvarado J. Goffinet J. Michael V. Liberti W. III Hatfield J. Gardner T. (2021). Neural dynamics underlying birdsong practice and performance. *Nature* 599 635–639. 10.1038/s41586-021-04004-1 34671166 PMC9118926

[B109] Sizemore M. Perkel D. J. (2008). Noradrenergic and GABA B receptor activation differentially modulate inputs to the premotor nucleus RA in zebra finches. *J. Neurophysiol.* 100 8–18. 10.1152/jn.01212.2007 18463188 PMC4071943

[B110] Sommer M. Koch M. A. Paulus W. Weiller C. Büchel C. (2002). Disconnection of speech-relevant brain areas in persistent developmental stuttering. *Lancet* 360 380–383. 10.1016/s0140-6736(02)09610-1 12241779

[B111] Tanaka M. Sun F. Li Y. Mooney R. (2018). A mesocortical dopamine circuit enables the cultural transmission of vocal behaviour. *Nature* 563 117–120. 10.1038/s41586-018-0636-7 30333629 PMC6219627

[B112] Ten Cate C. (2021). Re-evaluating vocal production learning in non-oscine birds. *Philos. Trans. R. Soc. Lond. B. Biol. Sci.* 376:20200249. 10.1098/rstb.2020.0249 34482726 PMC8419586

[B113] Ten Cate C. Fullagar P. J. (2021). Vocal imitations and production learning by Australian musk ducks (Biziura lobata). *Philos. Trans. R. Soc. Lond. B Biol. Sci.* 376:20200243. 10.1098/rstb.2020.0243 34482734 PMC8419576

[B114] Thoms G. Jürgens U. (1987). Common input of the cranial motor nuclei involved in phonation in squirrel monkey. *Exp. Neurol.* 95 85–99. 10.1016/0014-4886(87)90009-4 3792484

[B115] Tyack P. L. (2020). A taxonomy for vocal learning. *Philos. Trans. R. Soc. Lond. B Biol. Sci.* 375:20180406. 10.1098/rstb.2018.0406 31735157 PMC6895552

[B116] Veit L. Tian L. Y. Monroy Hernandez C. J. Brainard M. S. (2021). Songbirds can learn flexible contextual control over syllable sequencing. *Elife* 10:e61610. 10.7554/eLife.61610 34060473 PMC8169114

[B117] Vergne A. L. Pritz M. B. Mathevon N. (2009). Acoustic communication in crocodilians: from behaviour to brain. *Biol. Rev. Camb. Philos. Soc.* 84 391–411. 10.1111/j.1469-185X.2009.00079.x 19659884

[B118] Vernes S. C. (2017). What bats have to say about speech and language. *Psychon. Bull. Rev.* 24 111–117. 10.3758/s13423-016-1060-3 27368623 PMC5325843

[B119] Vicario D. S. (1994). Motor mechanisms relevant to auditory-vocal interactions in songbirds. *Brain Behav. Evol.* 44 265–278. 10.1159/000113581 7842285

[B120] Viceic D. Fornari E. Thiran J. P. Maeder P. P. Meuli R. Adriani M. (2006). Human auditory belt areas specialized in sound recognition: a functional magnetic resonance imaging study. *Neuroreport* 17 1659–1662. 10.1097/01.wnr.0000239962.75943.dd 17047449

[B121] Wang S. Liu S. Wang Q. Sun Y. Yao L. Li D. (2020). Dopamine modulates excitatory synaptic transmission by activating presynaptic D1-like dopamine receptors in the RA projection neurons of zebra finches. *Front. Cell Neurosci.* 14:126. 10.3389/fncel.2020.00126 32477072 PMC7235289

[B122] Wang S. Sun Y. Wang Q. Qiu Y. Yao L. Gong Y. (2019). Sexual dimorphism of inhibitory synaptic transmission in RA projection neurons of songbirds. *Neurosci. Lett.* 709:134377. 10.1016/j.neulet.2019.134377 31352043

[B123] White S. A. (2010). Genes and vocal learning. *Brain Lang.* 115 21–28. 10.1016/j.bandl.2009.10.002 19913899 PMC2888939

[B124] Wild J. M. Li D. Eagleton C. (1997). Projections of the dorsomedial nucleus of the intercollicular complex (DM) in relation to respiratory-vocal nuclei in the brainstem of pigeon (*Columba livia*) and zebra finch (Taeniopygia guttata). *J. Comp. Neurol.* 377 392–413. 10.1002/(sici)1096-9861(19970120)377:3<392::aid-cne7<3.0.co;2-y 8989654

[B125] Woolley S. C. (2016). Social context differentially modulates activity of two interneuron populations in an avian basal ganglia nucleus. *J. Neurophysiol.* 116 2831–2840. 10.1152/jn.00622.2016 27628208 PMC5168002

[B126] Woolley S. C. Rajan R. Joshua M. Doupe A. J. (2014). Emergence of context-dependent variability across a basal ganglia network. *Neuron* 82 208–223. 10.1016/j.neuron.2014.01.039 24698276 PMC4132189

[B127] Xiao L. Merullo D. P. Koch T. M. I. Cao M. Co M. Kulkarni A. (2021). Expression of FoxP2 in the basal ganglia regulates vocal motor sequences in the adult songbird. *Nat. Commun.* 12:2617. 10.1038/s41467-021-22918-2 33976169 PMC8113549

[B128] Yu A. C. Margoliash D. (1996). Temporal hierarchical control of singing in birds. *Science* 273 1871–1875. 10.1126/science.273.5283.1871 8791594

[B129] Zemel B. M. Nevue A. A. Dagostin A. Lovell P. V. Mello C. V. von Gersdorff H. (2021). Resurgent Na(+) currents promote ultrafast spiking in projection neurons that drive fine motor control. *Nat. Commun.* 12:6762. 10.1038/s41467-021-26521-3 34799550 PMC8604930

[B130] Zhang Y. Wang Q. Zheng Z. Sun Y. Niu Y. Li D. (2022). BDNF enhances electrophysiological activity and excitatory synaptic transmission of RA projection neurons in adult male zebra finches. *Brain Res.* 1801:148208. 10.1016/j.brainres.2022.148208 36549361

